# A review of cutting-edge biomarkers for diagnosing coronary artery disease

**DOI:** 10.1097/MD.0000000000041377

**Published:** 2025-01-24

**Authors:** Pouria Azami, Sahand Mohammadzadeh, Soroush Seirafi, Iman Razeghian-Jahromi

**Affiliations:** a Cardiovascular Research Center, Shiraz University of Medical Sciences, Shiraz, Iran; b Department of Pathology, Shiraz University of Medical Sciences, Shiraz, Iran; c Department of Cardiology, Shiraz University of Medical Sciences, Shiraz, Iran.

**Keywords:** biomarkers, coronary artery disease, diagnosis

## Abstract

Chronic coronary artery disease (CAD) remains a significant global healthcare burden. Current risk assessment methods have notable limitations in early detection and risk stratification. Hence, there is an urgent need for innovative biomarkers that facilitate the premature CAD diagnosis, ultimately leading to reduction in associated morbidity and mortality rates. This review comprehensively examines recent advances in emerging biomarkers for CAD detection. Our analysis delves into various aspects of these biomarkers such as their mechanisms of action, roles in the pathophysiology of the disease, and different measurement techniques employed in clinical practice. Comparative assessment of biomarker performance between CAD patients and control groups was also presented relying on their sensitivity, specificity, and area under the curve at specific cutoff points. In this regard, prominent biomarkers including Tenascin-C, IL-37, PTX3, transthyretin, soluble interleukin-6 receptor α, and miR-15a are identified as having high diagnostic potential for chronic CAD that indeed showcase promising performance metrics. These findings underscore the role of novel biomarkers in enhancing CAD risk stratification and improving patient outcomes through early intervention. However, the pursuit of an ideal and inclusive biomarker continues due to the multifaceted nature of CAD. Future randomized controlled trials are essential to bridge the gap between research findings and clinical practice in order to augment the practical application of these biomarkers in routine healthcare settings.

## 1. Introduction

Cardiovascular diseases (CVDs) are the leading cause of death globally. Coronary artery disease (CAD) is the most common form of CVD, which imposes the largest burden on the healthcare system.^[1]^ Growing incidence of premature CAD-related deaths,^[2]^ necessitates early diagnosis. In recent years, novel cardiac biomarkers have attracted great attention as they are known as representatives of biological and pathological changes. CAD is presented in various forms ranging from chronic coronary syndrome (CCS) to acute coronary syndrome (ACS) with different manifestations from unstable angina (UA) to life-threatening events.^[3]^ CAD exists along a continuum that includes both stable, chronic phases, and acute phases. ACS conditions, including UA, non-ST-elevation myocardial infarction, and ST-elevation myocardial infarction (STEMI), are characterized by acute myocardial injury, which can be identified by biomarkers like Troponins and CK-MB.^[4]^

The progression of CAD is a dynamic and unpredictable process that may result in major adverse cardiovascular events including revascularization, myocardial infarction (MI), stroke, and death.^[5]^ Coronary angiography is considered the gold standard for CAD diagnosis and is also a valuable method for reperfusion in the form of percutaneous coronary intervention. However, it is rarely used for early diagnosis due to its invasive nature, exposure to ionizing radiation, high cost, potential side effects, and an increased risk of complications like restenosis or thrombosis.^[6,7]^ Notably, atherosclerotic lesions preferentially grow outward within the layers of coronary arteries rather than inward. This makes diagnosis of CAD hard even during angiography. As a significant number of such atherosclerotic patients do not show symptoms of stenosis until the later stages, an acute MI may be inevitable.^[8]^

**Figure 1.**
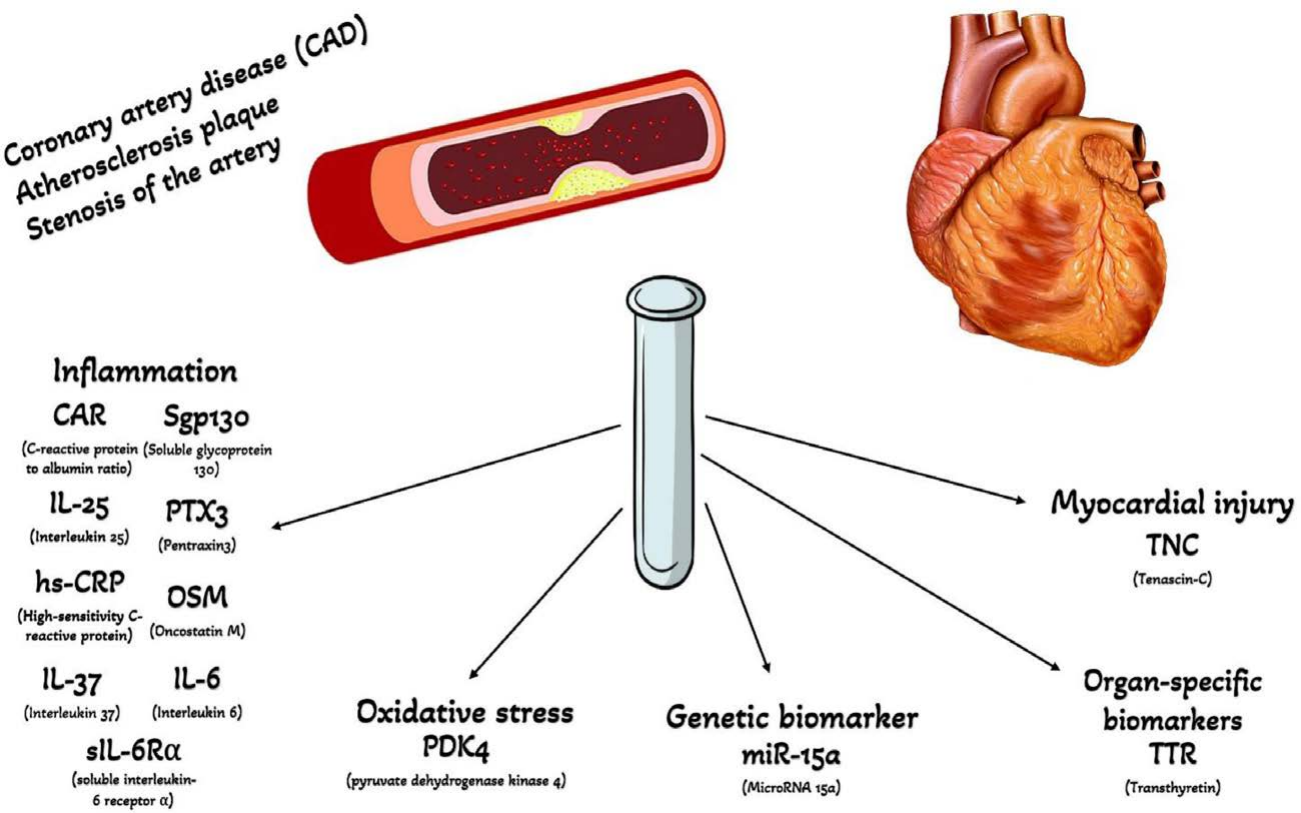
Biomarkers for diagnosis of coronary artery disease. CAD = coronary artery disease, CAR = C-reactive protein to albumin ratio, hs-CRP = high-sensitivity C-reactive protein, IL-6 = interleukin-6, IL-25 = interleukin-25, IL-37 = interleukin-37, miR-15a = microRNA 15a, OSM = Oncostatin M, PDK4 = pyruvate dehydrogenase kinase 4, PTX3 = Pentraxin 3, sgp130 = soluble glycoprotein 130, sIL-6Rα = soluble interleukin-6 receptor alpha, TNC = Tenascin-C, TTR = Transthyretin.

Relying on this background, identifying biomarkers involved in the initiation, development, and rupture of atherosclerotic plaques plays a critical role in reducing cardiovascular hospitalizations and mortality. Diagnosis of CAD with the aid of these biomarkers facilitates the implementation of targeted interventions to stop or even reverse the CAD progression. This review concentrates on novel biomarkers with high sensitivity and specificity for diagnosing CAD (Fig. 1).

## 2. Pathophysiology of CAD

CAD primarily originates from the growth of atherosclerotic plaques leading to the progressive narrowing of coronary arteries which, in turn, restricts blood supply to the myocardium and results in complications such as myocardial ischemia, hypoxia, and necrosis.^[9,10]^ Although the sophisticated process underlying the pathophysiology of atherosclerosis is not yet understood precisely, inflammation appears to be a fundamental component of plaque formation and development that greatly contributes to its local and systemic complications.^[5]^ Previously, atherosclerosis was thought to be a cholesterol storage disease; but now, it is believed that there is a complex interaction between blood and the arterial wall cells mediated by specific molecular players.^[8]^

## 3. Biomarkers for CAD diagnosis

Table 1 provides a concise summation of the findings extracted from the included articles. Our survey covers both general insights into these biomarkers and their specific implications in the context of atherosclerosis and CAD. Furthermore, we thoroughly review the primary methodologies and key findings outlined in the relevant articles.

**Table 1:** Diagnostic biomarkers of stable CAD.

Row	Studies, year	Country	Biomarker category	Study design	Main inclusion criteria	Laboratory test	No of patients/enrollment	Biomarkers’ concenteration	Findings	ROC curve (AUC, cutoff, sensitivity, specificity)	Reference
1	Gholipour, A. et al (2022)	Iran	TNC (Tenascin-C): biomarker of myocardial injury	Case–control	Patients with stable chest pain who underwent conventional coronary angiography	RNA-sequencing, qRT-PCR	All (n = 60) control group (n = 20), CAD group (n = 40)	TNC (Tenascin-C)	TNC expression plays a role in the differentiation of endothelial cells and the development of CAD, indicating its potential as a biomarker for the early detection of CAD.	AUC = 0.744 (95% confidence interval, 0.58–0.907; *P* = .011)	^[11]^
2	Manoochehri, M. et al (2021)	Iran	IL-25: biomarker of inflammation	Cross-sectional	Patients with an initial diagnosis of coronary artery disease who were undergoing angiography	ELISA	CAD (n = 64)	IL-25 [pg/mL] 24.87 ± 3.76 Other biomarkers: NHR (neutrophil to HDL-C ratio) [count.dL/mg] 2.52 ± 1.3 PHR (platelet-to-HDL-C ratio) [count.dL/mg] 2.52 ± 1.3 MHR (monocyte to HDL-C ratio) [count.dL/mg] 0.15 ± 0.1	IL-25 and MHR are valuable screening tools for cardiovascular disease risk. They also strongly indicate the severity of coronary artery disease, negatively correlating with the Gensini score and positively correlating with it, respectively.	–	^[7]^
3	Walter, J. et al (2019)	Switzerland	Interleukin-6 (IL-6): biomarker of inflammation	This study is a part of a large ongoing prospective diagnostic study	Patients with suspected functionally relevant CAD who underwent rest/ stress myocardial perfusion single-photon emission tomography/computer tomography (MPI-SPECT)	Micro-particle immunoassay	All patients (n = 1553), Functionally relevant CAD (n = 888), Not functionally relevant CAD (n = 665)	Interleukin-6 (IL-6) Functionally relevant CAD: 1.56 pg/mL Not functionally relevant CAD: 1.30 pg/mL	Patients with functionally relevant CAD exhibited elevated IL-6 levels compared to those without CAD. However, the diagnostic accuracy of IL-6 was generally moderate to low. Despite this, IL-6 still serves as a robust and independent predictor of cardiovascular death and all-cause mortality.	AUC = 0.57, 95% CI: 0.55–0.61	^[12]^
4	Ikeda, S. et al (2021)	Japan	Oncostatin M (OSM): biomarker of inflammation	Prospective cross-sectional study	Patients who underwent elective coronary angiography (CAG)	ELISA	All (n = 315), CAD positive (n = 169), CAD negative (n = 146),	Oncostatin M (OSM) pg/mL CAD positive: 123.0 ± 46.7 CAD negative: 98.3 ± 47.9	Serum OSM could be a novel biomarker for CAD detection and useful in screening asymptomatic CAD in DM patients.	AUC = 0.65 optimal cutoff: 120 pg/mL	^[13]^
5	Tong, L. et al (2019)	China	IL-37 (interleukin-37): anti-inflammatory biomarker PTX3 (pentraxin 3): inflammatory biomarker	Cohort	Inpatients suspected acute coronary syndrome who underwent coronary angiography	ELISA	All (n = 259), CAD (n = 180), control groups (n = 79),	IL-37 (interleukin-37) pg/mL CAD group 87.74 (72.14–108.28) Control group 185.38 (153.32–218.21), PTX3 (pentraxin3) ng/mL CAD group 3.93 (3.18–5.27) Control group 1.56 (0.55–2.34), Other biomarkers: RBP4 (retinol-binding protein-4) ng/mL CAD group 5.79 (4.49–6.96) Control group 3.60 (2.07–5.25), PAI-1 (plasminogen activator inhibitor-1) pg/mL CAD group 770.89 (719.27–851.58) Control group 709.79 (592.0–775.90), NTN1 (Netrin-1) pg/mL CAD group 50.45 (30.83–69.78) Control group 89.28 (60.08–126.26), GAL-3 (galectin-3) ng/mL CAD group 3.35 (2.10–4.47) Control group 1.14 (0.56–1.99), ADP (adiponectin) ng/mL CAD group 8.13 (6.88–9.21) Control group 11.24 (10.18–12.14),	Inflammatory adipocytokines, including retinol-binding protein-4 (RBP4), pentraxin 3 (PTX3), galectin-3 (GAL-3), and plasminogen activator inhibitor (PAI-1), exhibited elevated levels, while anti-inflammatory cytokines netrin-1 (NTN1), interleukin-37 (IL-37), and adiponectin (ADP) showed reduced levels in CAD patients compared to controls. Among these, PTX3 and IL-37 demonstrated the highest sensitivity and specificity, suggesting their potential as robust biomarkers for CAD diagnosis.	IL-37: AUC = 0.908, *P*-value = <.001, 95% CI = (0.863–0.952), sensitivity = 83.54%, specificity = 90.00%, cutoff (pg/mL) = 140.86. PTX3: AUC = 0.914, *P*-value = <.001, 95% CI = (0.875–0.943), sensitivity = 96.47%, specificity = 82.19%, cutoff (ng/mL) = 2.41.	^[14]^
6	Monu (2020)	India	Transthyretin (TTR): organ-specific biomarker	Cross-sectional study	CAD patients who were having multivessel stenosis, as affirmed by the coronary CT angiography	Two-dimensional gel electrophoresis (2-DE) and Protein Identification by MALDI-TOF, Western blot, ELISA, immunofluorescence (IF), fluorescence activated cell sorting (FACS), in silico analysis	All (n = 250), CAD patients (n = 200), Healthy individuals (n = 50)	TTR: Western Blot – 1.5-fold lower in CAD versus control, *P* < .023, ELISA – 1.61-fold lower in CAD vs. control, *P* < .0001, FACS – 2-fold lower in CAD vs. control, protein expression: 5.92% vs. 13.13% Other biomarkers: serotransferrin, talin-1, alpha-2HS glycoprotein, transthyretin (TTR), fibrinogen-α chain	Serotransferrin, talin-1, alpha-2HS glycoprotein, and transthyretin (TTR) were found to have lower level, whereas fibrinogen-α chain was found to have higher level in CAD plasma compared to healthy control. TTR may consider to serve as a tool for CAD screening and therapeutic target	TTR: AUC = 0.930, *P*-value < .05	^[15]^
7	Zhou, M. et al (2020)	China	sIL-6Rα (soluble interleukin-6 receptor α): Biomarker of inflammation	Case–control	Postmenopausal women who had symptoms of chest tightness and pain	ELISA	All (n = 336), Controls (n = 181), CAD (n = 155)	sgp130 (soluble glycoprotein 130) (ng/mL): Controls = 179.13 (152.03–204.76), CAD = 105.52 (87.79–121.58). Other biomarkers: IL-6 (interleukin-6) (pg/mL) Controls 5.94 (4.31–7.59), CAD 6.31 (4.92–10.07). sIL-6Rα (soluble interleukin-6 receptor α) (ng/mL) Controls 16.05 (13.28–19.33), CAD 17.90 (15.11–22.44) B/T ratio (binary/ternary complex ratio) Controls 1.46 (1.40–1.52), CAD 1.54 (1.48–1.62).	CAD patients showed higher IL-6, sIL-6Rα levels, and B/T ratio, but lower sgp130 levels than controls. sgp130 and B/T ratio could be valuable biomarkers for CAD diagnosis and assessing coronary stenosis severity in postmenopausal women.	sIL-6Rα: AUC = 0.957, 95% CI = (0.928–0.986), *P*-value = <.001, cutoff (ng/mL)= <136.01, sensitivity = 0.953%, specificity = 0.874%	^[16]^
8	Saleh, A.M. et al (2022)	Germany	Hs-CRP: biomarker of inflammation	Cohort	Patients with stable or low risk unstable angina pectoris which underwent coronary angiography	ELISA	All (n = 108) no CAD (n = 35) CAD (n = 73)	Hs-CRP (high-sensitivity C-reactive protein) mg/L no CAD 1.9 ± 1.5 CAD 3.2 ± 1.8	As an inflammatory marker, hs-CRP can be useful in the acute setting to rule out significant coronary artery disease (CAD), and it should be combined with other imaging modalities to enhance its sensitivity	–	^[17]^
9	Zheng, P.F. et al (2020)	China	TNF (tumor necrosis factor): biomarker of inflammation	Cross-sectional	Patients suffering from CAD enrolled the study. CAD was defined as significantly coronary artery stenosis (≥ 50%) in at least anyone of the 3 main coronary vessels or their main branches (branch diameter ≥ 2 mm)	RT-qPCR	All (n = 421), Control (n = 203), CAD (n = 218)	TNF (tumor necrosis factor): 3.35-fold higher in CAD versus control, *P*-value < .001, Other biomarkers: SOCS3 (suppressor of cytokine signaling 3), TNFAIP3, CXCL8 (C-X-C motif chemokine ligand 8).	CXCL8, TNF, and SOCS3 genes, recognized for their involvement in inflammation, could potentially function as diagnostic biomarkers for CAD	–	^[18]^
10	Du, P. et al (2021)	China	PDK4 (pyruvate dehydrogenase kinase 4): Biomarker of oxidative stress	Case–control	CAD patients who diagnosed by angiography (at least 1 coronary artery with a stenosis ≥ 50% of the diameter)	RNA-sequencing, qRT-PCR	All (n = 36), CAD (n = 18), Control (n = 18)	PDK4 (pyruvate dehydrogenase kinase 4): >3-fold higher in CAD versus control, *P* < .05	PDK4 may play an important role in abnormal activation of CD14 + monocytes in CAD patients. PDK4 may play a role in development of CAD	–	^[19]^
11	Pan, P. et al (2020)	China	miR-15a: Genetic biomarker	Cross-sectional	All the randomized patients were diagnosed with CAD by electrocardiogram (ECG), coronary calcium scoring or coronary CT angiography.	RT-qPCR	All (n = 172) CAD (n = 92) non-CAD (n = 80)	miR-15a (MicroRNA 15a) CAD 0.394 ± 0.258 non-CAD 1.152 ± 0.427	Lower expression of miR-15a in CAD patients and negative correlations with LDL-C, Gensini score, and inflammatory cytokines. MiR-15a holds potential as a prospective biomarker for the early detection of CAD	AUC = 0.9367, *P*-value = .0016	^[20]^
12	Erdöl, M.A. et al (2021)	Turkey	CAR (C-reactive protein to albumin ratio): Biomarkers of inflammation	Retrospectively cross-sectional	Patients with chest pain underwent coronary CTA	-	All (n = 187), CAC (coronary artery calcium) score = 0 (very low risk group) (n = 69), CAC score = 1–400 (low - moderate risk group) (n = 87), CAC score > 400 (high risk group) (n = 31)	CAR (C-reactive protein to albumin ratio) CAC score = 0, 0.61 (0.25–0.95); CAC score = 1–400, 0.71 (0.38–1.37); CAC score > 400, 1.57 (0.92–3.07)	An elevated CAR could serve as a predictive indicator for atherosclerosis and CAD. Utilizing CAR might prove valuable in the care and assessment of patients prior to undergoing invasive coronary angiography.	–	^[21]^
13	Tanriverdi, Z. et al (2020)	Turkey	CAR (C-reactive protein to albumin ratio): biomarkers of inflammation	Cross-sectional	Patients with stable angina pectoris who underwent CAG		All (stable angina pectoris): 421 Three-vessel disease (+), n = 53 Three-vessel disease (-), n = 368	CAR100: 3-vessel disease (+), (n = 53), 11.9 (3.3–24.1) 3-vessel disease (-), (n = 368), 3.3 (1.1–9.6) *P* < .001	CAR demonstrated the highest diagnostic effectiveness among the evaluated inflammatory parameters for detecting significant CAD	AUC of CAR was significantly higher than that of both CRP (0.819 vs 0.799, *P* < .001) and albumin (0.819 vs 0.681, *P* < .001)	^[22]^

AUC = area under the curve, CAC = coronary artery calcium, CAD = coronary artery disease, CAG = coronary angiography, CAR = C-reactive protein to albumin ratio, CBCT = computed tomography, CT = computed tomography, CTA = computed tomography angiography, CXCL8 = C-X-C Motif Chemokine Ligand 8, DM = diabetes mellitus, ECG = electrocardiogram, LDL-C = low-density lipoprotein cholesterol, miR-15a = MicroRNA 15a, PDK4 = pyruvate dehydrogenase kinase 4, qPCR = quantitative polymerase chain reaction, qRT-PCR = quantitative reverse transcription polymerase chain reaction, RNA-sequencing = RNA sequencing, SOCS3 = suppressor of cytokine signaling 3, TNF = tumor necrosis factor, TNFAIP3 = TNF Alpha-Induced Protein 3.

## 4. Interleukin-6 (IL-6)

IL-6 is a cytokine that plays an important role in various physiological processes. It is mainly produced from immune cells as well as nonimmune cells. During atherosclerotic plaque formation, activated immune cells produce inflammatory cytokines, which stimulate the activation of significant amounts of IL-6. IL-6 is the primary driver of C-reactive protein (CRP), serum amyloid A, and fibrinogen production.^[23]^ Circulating IL-6 is a predictor of CAD^[24,25]^ and cardiac events.^[26,27]^ In patients with CCS, elevated concentration of IL-6 is independent predictor of high-risk coronary anatomy.^[28]^

The association of this inflammatory biomarker with the incidence and severity of subclinical atherosclerosis in patients with nonalcoholic fatty liver disease has been investigated. Notably, IL-6 was the only biomarker found to be associated with coronary artery calcium (CAC) scores.^[29]^ Also, IL-6 greatly contributes to atherosclerosis progression predicting plaque growth in the carotid artery.^[30]^ IL-6 was independently associated with the risk of cardiovascular death, major adverse cardiovascular events, MI, hospitalization due to heart failure, and all-cause mortality. This confirms the undeniable role of IL-6 in the inflammatory processes and cardiovascular events.^[27,31]^ IL-6 improves the prediction of CAD incidence in patients who have intermediate cardiovascular risk. In 1 study, all the patients with IL-6 of ≥ 1.0 pg/mL suffered from CAD, while only 64% of patients with IL-6 < 1.0 pg/mL showed CAD symptoms. They suggested that IL-6 is a valuable determinant in categorizing patients with intermediate atherosclerotic CVD risk.^[32]^ In a recent study, Walter et al declared that IL-6 concentrations were significantly higher in functionally relevant CAD than in healthy controls. Despite this evident association, its clinical usefulness is limited due to low to moderate accuracy. After adjusting for confounding variables, IL-6 levels were not found to be an independent predictor of functionally relevant CAD. However, this inflammatory biomarker may remain as a predictor for cardiovascular death and all-cause death during long-term follow-up.^[33]^ The association between IL-6 and CAC has been demonstrated in multiple studies.^[12,34]^ Specifically, higher IL-6 levels correlate with elevated CAC scores and progression of atherosclerosis, suggesting that IL-6 may be a potential biomarker for CAD and a marker of subclinical CVD.^[13]^

## 5. Interleukin-25 (IL-25)

IL-25 (IL-17 E) is a member of the IL-17 family that distinctly differs from other family members structurally and biologically.^[35]^ IL-25 is primarily produced by TH2 cells besides a variety of other cells.^[36–38]^ IL-25 levels are usually determined by the ELISA method on serum samples of peripheral blood. It is crucial in modulating immune responses. This mediator induces and enhances Th2 immune responses associated with allergic disorders. The mechanisms through which IL-25 affects Th2 immune response involve stimulation of inherent lymphoid type 2 cells to produce high levels of certain cytokines along with induction of low levels of Th2 cell differentiation in the presence of CD4^+^ T cells.^[39,40]^ Moreover, IL-25 inhibits Th1/Th17-mediated immune responses.^[41]^ Noteworthy, it is expressed by both healthy and atherosclerotic arteries. It is also found in smooth muscle cells, endothelial cells, and mature B cells, especially in developed plaques. Simply, the presence of IL-17E in atherosclerosis components indicates its sizeable involvement in this complex process.

In a recent study, authors reported that as the severity of stenosis increased, the concentration of IL-25 decreased while the concentrations of neutrophil to HDL-C ratio (NHR) and monocyte to HDL-C ratio (MHR) increased. The findings also revealed a negative correlation between IL-25 and the Gensini score. They concluded that IL-25 has an atheroprotective role in patients with CAD. Also, MHR and NHR positively correlated with the Gensini score.^[7]^ However, Xu et al paradoxically indicated that IL-25 is significantly higher in CAD patients, especially in those who experienced AMI, compared with the healthy control. Also, they claimed a positive correlation between IL-25 and the Gensini score.

## 6. Soluble glycoprotein 130 (gp130)

The membrane-bound beta IL-6 receptor, also known as glycoprotein 130 (gp130), is a pivotal component of the IL-6 trans-signaling pathway. Its involvement in the interaction between IL-6 and the soluble form of the membrane-bound alpha IL-6 receptor (sIL-6Rα) results in an inflammatory cascade associated with atherosclerosis.^[42]^ The cleavage of the membrane-bound gp130 receptor releases a soluble form of gp130 into the bloodstream.^[43]^ Furthermore, elevated concentrations of sgp130 have been proposed to block the classic signaling pathway by sequestering free IL-6 into IL-6:IL-6Rα:sgp130 complexes. This inhibition prevents IL-6 from efficiently binding to the membrane-bound IL-6 receptor and initiating classic signaling.^[44]^ As a result, sgp130 exerts a protective effect by impeding the inflammatory cascade involved in atherosclerosis, and thereby, sgp130Fc, a combination of sgp130 and IgG1-Fc, holds promise as a potential treatment for atherosclerosis. It demonstrated outstanding therapeutic potential by effectively attenuating atherosclerosis and causing significant regression of severe atherosclerosis in hypercholesterolemic mice models.^[45]^ Endothelial activation, smooth muscle cell infiltration, and monocyte recruitment were also reduced, and atherosclerotic plaque progression was remarkably decreased accordingly.

sgp130 has been identified as a promising biomarker for diagnosing and assessing the severity of CAD. The sgp130 level was significantly lower compared with the control group. Also, a negative correlation was found between sgp130 levels and the Gensini score, in which a higher sgp130 level was identified as the sole predictor of lower risk of CAD incidence in postmenopausal women. Furthermore, they defined a cutoff value for the sgp130 level at 136.01 ng/mL. Moreover, the study demonstrated that elevated level of IL-6 was associated with higher Gensini scores, and CAD patients exhibited higher concentrations of this biomarker and sIL-6Rα compared with healthy postmenopausal women.^[46]^ In a separate investigation, researchers found that individuals with chronic or acute CAD showed notably lower levels of sgp130 in comparison to the control subjects without CAD. Also, patients with acute CAD and a 2- or 3-vessel CAD had even further reduced sgp130 levels than those with chronic CAD and only 1-vessel disease.^[45]^

## 7. Tumor necrosis factor (TNF)

TNF is a proinflammatory cytokine that belongs to the TNF superfamily and is primarily produced by immune cells. It plays an important role in various biological processes, and contributes to chronic inflammation and atherosclerosis.^[16]^ TNF promotes endothelial dysfunction in blood vessels by triggering the production of adhesion molecules and chemokines within the endothelial cells, which in turn attract other immune cells such as monocytes. The adhered monocytes transform into macrophages and ultimately lead to the formation of atherosclerotic plaques.^[47]^ Furthermore, TNF has been involved in the induction of proliferative and angiogenic factors.^[16]^ Metabolic dysregulation, including hyperglycemia, dyslipidemia, and adiposity elevates TNF levels.^[48]^ In experimental studies, TNF-deficient mice had reduced plaque size.^[49]^ Increased TNF level has been linked to atherogenesis and associated with higher rates of ischemic events, and it has also been related to cardiovascular prognosis.^[50–52]^ In particular, TNF level serves as a strong predictor of recurrent events after MI.^[53]^ Several other studies have also indicated that anti-TNF treatment reduces the incidence of atherosclerosis and cardiovascular sequels.^[54]^

TNF stands out as a key component since its alteration may serve as a potential diagnostic biomarker.^[55–57]^ A relationship between specific TNF polymorphisms and CAD was reported.^[58]^ One study identified 20 upregulated differentially expressed genes from 2 CAD and 1 ischemic stroke (IS) patients. Noteworthy, the Molecular Complex Detection (MCODE) analysis revealed a combination of the top 5 hub genes: JUN, CXCL8, TNF, SOCS3, and TNFAIP3. Further analysis confirmed CXCL8 upregulation in IS patients as well as increased expression of SOCS3, TNF, and TNFAIP3 in both CAD and IS patients. The study found that TNF expression in CAD patients was approximately 3.35 times higher than in healthy individuals. Furthermore, unconditional logistic regression analysis revealed a significant correlation between the expression levels of CXCL8, SOCS3, TNF, and TNFAIP3 and the incidence of CAD and IS.^[59]^

## 8. Tenascin-C (TNC)

TNC is a remarkable extracellular glycoprotein from the tenascin gene family that is predominantly expressed during embryonic development. This matrix component may have a significant effect on tissue remodeling, cell migration, proliferation, and apoptosis.^[60]^ TNC is a prognostic biomarker in a variety of inflammatory conditions.^[18,61]^ TNC reemerges in many myocardial pathologic conditions. Upregulation of TNC is observed in myocarditis,^[61]^ atherosclerotic plaques, MI,^[62,63]^ and pulmonary hypertension^[64]^ underscoring its sensitivity in detecting early cell injury. It is believed that this molecule plays a fundamental role in cardiomyocyte survival in the early steps of MI by weakening the strong adhesion to connective tissue. Also, TNC facilitates myocardial tissue remodeling by influencing interstitial cell behavior during post-injury tissue repair.^[63]^

TNC expression promotes certain changes in smooth muscle cells from a nonproliferative phenotype to a migratory synthetic state leading to plaque growth. TNC also forms a positive feedback loop with matrix metalloproteinases that augments plaque progression, destabilization, and rupture. Moreover, this player provides adhesive surfaces for glycoprotein receptors on platelets, which facilitates thrombus formation in advanced atherosclerosis.^[65]^ TNC was identified as a promising noninvasive biomarker for CAD diagnosis. TNC showed differential expression in CAD patients compared with non-CAD individuals. They also measured the expression level of TNC during endothelial cell transition; their analysis showed gradual upregulation from human embryonic stem cells to early vascular progenitor cells, followed by a decrease until endothelial cell maturation.^[66]^ A TNC cutoff of 100.91 ng/mL predicted a higher Gensini score.^[65]^ Higher concentrations of TNC in CAD patients suggesting its potential as a diagnostic marker.^[67]^

The relationship between TNC and coronary calcium scores has been studied. In 2 studies, elevated serum TNC levels were attributed with higher score suggesting that TNC may be a useful biomarker for assessing the severity of coronary atherosclerosis. In 1 study, a positive correlation was observed between TNC and the Gensini score.^[65]^ Similarly, association of TNC levels with higher calcium scores indicates a potential role for TNC in early CAD detection and risk stratification.^[68]^

## 9. Pyruvate dehydrogenase kinase 4 (PDK4)

PDK4, a member of the PDK family, is an enzyme involved in glucose metabolism by regulating the pyruvate dehydrogenase complex (PDC).^[69]^ PDK4 is primarily expressed in tissues with high metabolic activity such as heart.^[11]^ Also, it is linked to diabetes, obesity, and insulin resistance, which are all risk factors of cardiovascular disorders.^[70]^ The extensive research conducted on PDK4 underscores its significance as a potential therapeutic target for addressing these metabolic disorders.^[71]^

During chronic inflammation, PDK4 expression rises, which is mediated by the phosphorylation of Jun N-Terminal Kinases.^[72]^ Moreover, regulation of E2F1-dependent PDK4 expression by NF-κB is crucial for maintaining cardiac health.^[73]^ Although PDK4 significantly regulates cardiac energy metabolism, there is no conclusive evidence for a direct link between PDK4 and CAD. However, certain studies have shown its contribution to the modulation of cardiac energy metabolism is particularly important during stress conditions, which increases the risk of heart failure.^[74]^

It has been reported that PDK4 is a downstream target of miR-148 in the context of myocardial ischemia-reperfusion injury. PDK4 inhibition not only reduced cardiomyocyte apoptosis but also improved cardiac function and immune responses. These findings highlight the therapeutic potential of targeting PDK4 to address the multifaceted challenges associated with ischemic heart disease.^[75]^ It also promotes vascular calcification by disrupting autophagy and shifting vascular smooth muscle cells (VSMCs) toward glycolysis, which reduces mitochondrial function and leads to calcium buildup. This process contributes to calcium accumulation in vascular cells and an increase in CAC scores.^[76]^

Du et al identified 753 upregulated and 2144 downregulated genes among 2897 differentially expressed genes in CD14^+^ monocytes from CAD patients. To validate these findings, 10 genes were selected for further analysis. Among the upregulated genes, the mRNA expression levels of FOSL2, Oncostatin M (OSM), PFKFB3, and PDK4 were significantly higher. For the downregulated genes, the mRNA expression levels of RHOB, PTGER4, and DENND2D were significantly lower. Moreover, the study provided evidence that PDK4 is significantly overexpressed in LDL-exposed CD14^+^ monocytes indicating its potential role in the aberrant activation of CD14^+^ monocytes, which may be implicated in the pathogenesis of CAD. Hence, a promising potential is assumed for this biomarker for CAD diagnosis.^[10]^

## 10. OSM

OSM is a glycoprotein (gp130) that belongs to IL-6 family cytokines,^[77]^ and is secreted from immune cells and several other cell types.^[78]^ Members of this family are involved in immune homeostasis, hematopoiesis, inflammation, development, metabolism, reproduction, tissue regeneration, protection, and repair against infection, trauma, or injury.^[79]^ Adipose tissue-derived OSM plays a role in metabolic diseases, and is elevated in obesity and type 2 diabetes mellitus.^[77]^ The JAK3/STAT3 signaling pathway is the primary mechanism through which OSM promotes the differentiation of human vascular smooth muscle cells into osteoblasts. As a result, OSM that originates from macrophages during atherosclerosis is an important factor in advancing plaque calcification.^[19]^

Macrophage-derived OSM is also essential for cardiomyocyte proliferation. Activation of OSM co-receptor OSMR/gp130 enhances the proliferation of the cardiomyocytes.^[80]^ Zhang et al demonstrated the therapeutic potential of OSM in a mouse model of MI. OSM increased cardiac function and decreased apoptosis and fibrosis. It promotes angiogenesis by upregulating vascular endothelial growth factor and basic fibroblast growth factor in the infarct border zone of the ischemic myocardium.^[78]^ Gwechenberger et al confirmed that OSM contributes to cardiomyocyte repair via the cardiac reperfusion process following ischemic injury.^[81]^ Poling et al reported that attenuation of the OSM/receptor (Oβ) signaling pathway might inhibit the development of a mouse model of inflammatory dilated cardiomyopathy.^[82]^ However, this attenuation leads to deterioration of heart function after MI.^[83]^ OSM also induces angiogenesis by producing angiogenic factors.^[78]^ The presence of OSM promotes atherosclerotic plaque destabilization, and deficiency of the OSM receptor β may reduce plaque vulnerability.^[84]^

OSM was introduced as a diagnostic biomarker for CAD, and its value was declared as a screening tool for silent myocardial ischemia following diabetes mellitus. Serum level of OSM was significantly higher in patients with coronary organic stenosis compared with healthy peers. OSM levels was lower in restenosis condition in comparison to primary one-vessel stenosis in CAD patients.^[34]^ Furthermore, the association between elevated serum OSM levels and CAC suggests that OSM may play a role in developing atherosclerotic lesions, particularly in diabetes mellitus patients. Higher OSM levels have been shown to correlate with more severe coronary stenosis and increased CAC scores, further indicating its potential as a biomarker for CAD assessment.^[34]^

## 11. miRNA15

MicroRNAs (miRNAs) are small single-stranded RNAs that do not encode for protein synthesis. Their primary function is to regulate gene expression at the posttranscriptional level. They also act as either oncogenes or tumor suppressors, altering tumor cell proliferation, invasion, and apoptosis.^[85]^ Researchers have used microRNAs as biomarkers for the diagnosis of different pathologic conditions.^[86]^ For instance, miR-15a modulates coactivator-associated arginine methyltransferase 1 expression, which increases plaque susceptibility.^[87]^

MicroRNAs are also potent diagnostic biomarkers in CVD, especially CAD. miR-23 was found to be efficient in CAD patients for predicting the severity of the disease. It binds to vascular endothelial growth factor mRNA and suppresses its expression, which in turn decreases the angiogenic activity of endothelial progenitor cells (EPC).^[88]^ miR-574-5p is also elevated in CAD patients. It promotes VSMC proliferation and inhibits apoptosis by targeting the ZDHHC14 gene; thus, it is considered a potential molecular target for CAD treatment.^[89]^ MiR-183-5p distinguishes non-ST-elevation myocardial infarction from STEMI, CCS, and healthy individuals while miR-134-5p distinguishes STEMI patients from healthy individuals. A combined panel of miR-134-5p, miR-15a-5p, and let-7i-5p shows improved discrimination of STEMI patients from healthy controls.^[90]^

Certain miRNAs have anti-inflammatory effects. For example, miR-155 reduced the expression of TNF-α and IL-6 secreted by ox-LDL-induced macrophages.^[91]^ Plasma concentrations of miR-221, miR-130a, and miR-155 are lower in CAD patients. MiR-130a potentially serves as an independent indicator for predicting the occurrence of CAD.^[92]^ The expression level of miR-15a in peripheral blood samples was significantly lower in patients with CAD compared with non-CAD controls. The study suggested that miR-15a could serve as a novel diagnostic biomarker for CAD. Furthermore, lower miR-15a levels were associated with increased LDL-C, higher Gensini scores, and elevated inflammatory cytokines indicating its potential role in CAD diagnosis.^[93]^ miR-15a is also identified as a potential biomarker for vascular calcification in hemodialysis patients and CAD. In hemodialysis patients, elevated serum miR-15a, along with age and WBC count, predicts cardiovascular risk especially in coronary arteries with high diagnostic accuracy.^[94]^

## 12. Transthyretin (TTR)

TTR, as a homotetrameric protein, is predominantly synthesized by the liver^[95]^ and choroid plexus^[96]^ in the brain. TTR possesses extra functions including proteolytic activity and involvement in a variety of disorders such as cardiac amyloidosis.^[97]^ Evidence suggests an association between TTR and cardiovascular disorders. Low serum TTR levels upon admission have been found to independently predict major adverse cardiac events in patients with ACS during hospital stay.^[20]^ Altered expression of TTR has also been declared in carotid atherosclerotic lesions.^[98]^ It is associated with epicardial CAD and heart failure with preserved ejection fraction.^[99,100]^

The exact role of TTR in CAD pathogenesis is not fully understood. However, it is believed that it interacts with the apolipoprotein family implicated in cholesterol transport contributing to the development of CAD.^[101,102]^ The presence of TTR in different cell types and its altered level proposes its potential contribution to CAD. TTR secretes a signal peptide that facilitates amyloid formation. Through HDL- or LDL-mediated interaction with apolipoprotein A-I (Apo-A1), TTR significantly affects cholesterol efflux leading to amyloid deposition and cytotoxicity.^[101,103]^ These structural changes in TTR result in extracellular protein deposition in cardiac tissues and coronary arteries ultimately altering TTR level and its functioning during CAD.

Among 19 proteins examined in the CAD patients, 5 were differentially expressed in CAD plasma; serotransferrin, talin-1, alpha-2HS glycoprotein, and TTR. Findings indicated significant decreased expression in CAD patients. Furthermore, ELISA showed a 1.61-fold decline in TTR levels in CAD group. The flow cytometry analysis revealed a significant 2-fold decrease in TTR level in CAD plasma along with a decreased protein expression. The authors suggested that the lower level is a potential risk marker in CAD patients highlighting its importance as a predictive tool for disease severity and screening.^[104]^ In transthyretin amyloidosis, microcalcifications form in the heart contributing to tracer binding seen in bone scans. These tiny calcifications are distinct from coronary artery calcifications and are associated with cellular breakdown like autophagy rather than amyloid fibrils. This unique calcification pattern aids in diagnosing ATTR amyloidosis but does not reflect coronary calcium or total amyloid load.^[105]^

## 13. CRP to albumin ratio (CAR)

The CRP to albumin ratio (CAR) is a recently emerged inflammatory parameter that holds great promise in diagnosis and managing various disorders. CRP is a well-established biomarker primarily triggered by IL-6 with outstanding properties in inflammatory processes like atherosclerosis.^[106]^ Several studies have focused on its predictive value for CAD diagnosis and prognosis in individuals at high risk of MI.^[15,107,108]^ Conversely, albumin is a nutritional biomarker and the most abundant circulating protein in the plasma.^[109]^ It serves as a cardiovascular risk indicator and is implicated in atherogenesis and atherothrombosis.^[110]^ Notably, low levels of albumin have been associated with persistent systemic inflammation.^[111]^ A low serum level of albumin is inversely associated with long-term mortality, the progression of advanced heart failure in STEMI patients, and increased CAD severity in ACS patients.^[112,113]^

The CAR–CAD is suggested to predict CAD more effectively than CRP and albumin alone.^[21,114–116]^ It predicts in-stent restenosis following iliac stent implantation,^[117]^ serves as an independent predictor of all-cause mortality in STEMI,^[118]^ is correlated with the presence and severity of coronary artery ectasia,^[119]^ and when combined with the GRACE score, anticipates short-term major adverse cardiac events in percutaneous coronary intervention-treated STEMI patients.^[120]^

In a recent study,^[121]^ the authors found that CAR had the highest diagnostic accuracy for CCS compared to other markers such as the neutrophil-to-lymphocyte ratio, monocyte-to-lymphocyte ratio, mean corpuscular volume, HDL-C, and mean platelet volume to lymphocyte ratio. Moreover, CAR and age were identified as the only independent predictors of significant CAD among all the parameters studied.

In another study, it was demonstrated that the CAR predicts atherosclerosis and is valuable for CAD diagnosis, outperforming those of CRP and albumin individually. Moreover, the study showed a positive correlation between CAR level and both the CAC score and CAD-RADS score. These results suggest that CAR could be a promising biomarker for assessing the presence of atherosclerosis and CAD. It may aid in risk stratification and decision-making before invasive coronary angiography.^[109]^

## 14. Hs-CRP

CRP is a well-known acute-phase protein primarily produced by hepatocytes. Acute-phase proteins are increased in response to tissue damage, inflammation, infection, and malignancies.^[122]^ Its association with metabolic conditions like dyslipidemia, obesity, hypertension, insulin resistance, and type 2 diabetes has been confirmed previously.^[123]^ Clinically, CRP is widely used for screening organic diseases, assessing disease activity, classification of inflammatory disorders, and controlling infections.^[124]^ CRP levels are stable throughout the day and are not acutely affected by dietary changes.

Risk factors of atherosclerosis trigger proinflammatory cytokines. These cytokines, especially IL-6, prompt hs-CRP production by hepatocytes. CRP actively contributes to atheromatous lesions and plaque rupture and increases the expression of adhesion molecules and monocyte chemoattractant protein-1, provokes LDL uptake by macrophages, activates monocytes, induces a procoagulant effect, activates the complement pathway, and colocalizes with complement complexes in the coronary plaques.^[107]^

Previous studies have demonstrated an association of hs-CRP with mortality risk.^[22,125]^ Elevated hs-CRP independently predicts all-cause and cardiovascular mortality.^[124]^ hs-CRP also predicts all-cause and cardiovascular mortality and serves as an inflammatory marker related to atheroma plaque vulnerability.^[126,127]^ Hs-CRP also performs as a biomarker for CAD and major cardiovascular events irrespective of CAD severity. It is an inflammatory marker with a close relation to the vulnerability of the atheroma’s plaque.^[107]^ Individuals with low hs-CRP levels showed a reduced risk of developing stroke, coronary heart disease (CHD), and CHD-related death.^[128]^ Elevated levels of CRP were shown in patients with ACS.^[129,130]^

In a 2022 study, investigators identified hs-CRP as a measurable biomarker for detecting coronary stenosis.^[131]^ It was indicated that hs-CRP correlates with echocardiographic parameters both at rest and under dobutamine stress. Among various inflammation and oxidative stress biomarkers, hs-CRP stands out for its reproducibility and clinical practicality.^[132]^ Some studies have suggested a positive relationship between CRP levels and CAC.^[133,134]^ However, a more recent meta-analysis found no significant association between CRP and CAC. The pooled odds ratios suggest that CRP may not serve as a reliable prognostic marker for CAC when considered alongside traditional cardiovascular risk factors.^[135]^

## 15. Pentraxin 3 (PTX3) and interleukin-37 (IL-37)

PTX3 is a long multimeric acute-phase protein associated with CAD.^[17]^ It is released^[136]^ in response to tissue damage and inflammatory cytokines activation.^[17]^ PTX3 is known as an activator of the complement system and the humoral arm of the innate immune system.^[137]^ Consequently, it serves as a specific marker for inflammatory and atherosclerotic changes in the vascular wall^[138,139]^ independent of traditional risk factors.^[140]^ PTX3 is associated with advanced atherosclerosis and demonstrates superior predictive ability for CVD compared with CRP due to its higher specificity. However, it may not be effective in predicting early-stage atherosclerosis.^[136]^ PTX3 levels are increased in the early phase of AMI. Released by neutrophils, PTX3 binds to activated platelets leading to anti-inflammatory and antithrombotic effects, and possibly provides a cardioprotective effect against AMI.^[17]^ Elevated levels of PTX3 have been attributed to UA,^[141]^ adverse outcomes after MI,^[142]^ and heart failure,^[143]^ making it a promising indicator of vascular inflammation.^[144]^ PTX3 is also associated with cardiovascular mortality and all-cause mortality in patients with high risk of ACS.^[145]^ It is reported that a combination of Eicosapentaenoic acid and statin therapy reduces the PTX3 level and stabilizes atherosclerotic plaques in CCS.^[146]^

IL-37 is another novel anti-inflammatory marker belonging to the IL-1 family. It is primarily produced by peripheral blood mononuclear cells,^[147]^ and exhibits immunomodulatory effects. In particular, IL-37 reduces inflammatory cytokines and effectively inhibits inflammation and immune responses.^[148]^ This is achieved through suppression of proinflammatory cytokine synthesis, modulation of transcriptional cytokine expression, and prevention of kinase signaling activation.^[149,150]^ Notably, footprints of IL-37 are observed in atherosclerosis. It is expressed in foam-like cells underscoring its role in disease development.^[151]^ In ACS, a high concentration of IL-37 exerts a protective effect against atherosclerosis progression. However, elevated IL-37 level upon admission is associated with a poor prognosis.^[152]^

Furthermore, IL-37 is characterized as a possible predictor of severe CAC due to its association with elevated levels of coronary calcium.^[153]^ It interacts with the IL-1R8 receptor to directly mitigate platelet activation, thrombus formation, and myocardial injury.^[153]^ Studies have demonstrated significantly increased levels of IL-37, both in the plasma and within the coronary plaques, in mouse models of atherosclerotic disease, which were further attenuated by atorvastatin therapy.^[151]^

A group of proinflammatory and anti-inflammatory adipocytokines were studied in ACS patients.^[154]^ Notably, these biomarkers were significantly correlated with the Gensini score. Moreover, IL-37 was significantly downregulated in three-vessel CAD patients compared with the less severe CAD subgroups. Among inflammatory cytokines, PTX3 had the highest sensitivity and specificity. On the other hand, IL-37 showed highest specificity. It is suggested that a combination of these adipocytokines provides the highest specificity and sensitivity for CAD diagnosis and prognosis.

To assess CAC, PTX3 levels are increasingly recognized as an extra marker for cardiovascular risk stratification. PTX3 is associated with CAC indicating its role in identifying subclinical CVD.^[155]^ Moreover, PTX3 has been linked to the severity of CAD in specific populations such as hemodialysis patients,^[156]^ and predicted cardiovascular outcomes including mortality.^[157]^ Combining PTX3 with CAC enhances the noninvasive detection of CAD and improves early diagnosis and risk assessment.^[158]^ Similarly, IL-37, an anti-inflammatory cytokine, has been linked to CAC and atherosclerosis in both human and animal models. In patients with severe CAC, IL-37 levels were significantly elevated, representing a potential future biomarker for severe CAC.^[153]^ In diabetic mice, IL-37 treatment attenuated vascular calcification and atherosclerosis progression with its effects partially mediated by osteoprotegerin.^[159]^ These findings suggest IL-37 nomination as a therapeutic target for preventing or treating atherosclerotic diseases and vascular calcification.

## 16. Discussion

Many individuals with progressive CAD may not have traditional risk factors or symptoms. This necessitates the development of more comprehensive diagnostic algorithms to identify high-risk patients. The inclusion of exclusive biomarkers that aid discrimination of CAD from non-CAD individuals is crucial. Numerous biomarkers with diverse biological activities have been implicated in CAD progression. Given the multifactorial nature of CAD pathophysiology, attributing the entire disease process to a single biomarker is far from the reality. Therefore, a more sensible approach would be to consider the use of multi-biomarker panels. Each biomarker in such panels captures different aspects of CAD pathology. For instance, Eapen et al proposed an aggregate risk score consisting of CRP, fibrin degradation products, and heat shock protein 70 (HSP70) as a predictor for future risk of death and MI in patients with suspected or known CAD. Kleber et al introduced the VILCAD risk score, which includes age, sex, left ventricular ejection fraction, heart rate, N-terminal pro-brain natriuretic peptide (NT-proBNP), cystatin C, renin, 25-hydroxy-vitamin D3, and HbA1c for classifying and managing patients with CCS.^[14,160]^

Besides the routine use of anticoagulant and anti-lipid medications, there is growing interest in exploring the potential of new anti-inflammatory drugs in the context of CVD. One such approach involves targeting interleukin-1β (IL-1β) by monoclonal antibodies, such as Canakinumab, which may reduce cardiovascular events in patients with a history of AMI.^[161]^ Indeed, emerging evidence suggests that low-dose colchicine may have benefits for patients with atherosclerosis by reducing the risk of recurrent cardiovascular events. Colchicine exerts anti-inflammatory effects by blocking the NLRP3 inflammasome, which is responsible for the production of IL-1β and IL-18.^[162]^ These findings highlight the significance of inflammatory biomarkers in the development of CAD and propose their potential as promising tools in both diagnosis and prognosis. Moreover, targeting inflammatory pathways offers novel avenues for improving patient outcomes and controlling CAD more effectively.

Genomic and proteomic approaches, along with the utilization of artificial intelligence and machine learning algorithms, have allowed for the identification of previously overlooked molecular signatures associated with CAD. This presents an exciting opportunity for the development of next-generation biomarker panels that offer greater accuracy in detecting the preliminary stages of atherosclerotic plaque formation. Breakthroughs in high-throughput assay methods now empower researchers to measure a vast array of biomarkers. For instance, cutting-edge multiplex immunoassay panels allow for the simultaneous analysis of an extensive array of 92 protein biomarkers relevant to CVD. Also, multiplex technology has made it feasible to probe the proteome through aptamers capable of binding to proteins.^[163,164]^ Such risk panels predict the time to the first cardiac event more accurately facilitating premature detection and risk assessment for CCS.

Although this review has focused on CCS, biomarkers used in ACS remain essential for assessing the full CAD spectrum. ACS biomarkers like troponins and CK-MB are particularly relevant in detecting acute myocardial injury and are integral to ACS diagnosis. Inflammatory markers such as CRP and PTX3 are involved in both chronic and acute CAD phases highlighting the continuous nature of CAD pathophysiology.^[4]^ Including ACS biomarkers in CAD risk assessment may enhance monitoring for patients at risk of progressing from chronic CAD to acute events. The inflammatory processes underlying both ACS and CAD progression suggest that certain biomarkers may act as early indicators for patients at higher risk of transitioning to ACS.^[165]^ Future research should aim to develop multi-biomarker panels that address acute CAD as well. This approach may lead to more effective risk stratification strategies and targeted early interventions across the CAD continuum.^[166]^

Some of the mentioned biomarkers have also shown potential diagnostic and prognostic utility in related cardiovascular conditions such as IS, transient ischemic attack (TIA), and peripheral arterial disease. For IS and TIA, studies have found that inflammatory and pro-thrombotic markers including CRP and extracellular vehicles from platelets and leukocytes play critical roles in the pathophysiology by contributing to platelet activation and coagulation pathways.^[167,168]^ Specific microRNAs (e.g., miR-15a-3p, let-7f) have also been identified as promising biomarkers for early diagnosis of IS demonstrating a high predictive value for stroke severity and recurrence.^[167,169]^ In a review, diagnostic, and prognostic blood biomarkers in TIA and minor IS were evaluated, and key inflammatory biomarkers were reported as IL-6, IL-25, IL-37, TNF-α, and hs-CRP while S100 calcium-binding protein B (S100B), Neuron-specific Enolase, and Heart-type Fatty Acid-Binding Protein attributed to neuronal injury. Plasminogen Activator Inhibitor-1, von Willebrand Factor, and D-dimer show thrombosis whereas NT-proBNP reflects cardiac stress. Moreover, microRNAs (195-5p, miR-451a) and Neutrophil Gelatinase-Associated Lipocalin offer promising potential for improving diagnosis, prognosis, and risk stratification.^[168]^

In peripheral arterial disease, inflammatory biomarkers, and matrix metalloproteinases have shown to be implicated in disease progression. Noncoding RNAs and extracellular vehicles s may further aid in patient stratification and risk assessment.^[170,171]^ Biomarkers of ischemia-reperfusion injury such as lactate, CK, and myoglobin have shown particular relevance in acute limb ischemia, and possibly assist early identification of patients at risk of complications like rhabdomyolysis and kidney injury.^[172]^ While a detailed analysis of these conditions is beyond this review’s scope, the shared role of these biomarkers across various CVD underscores their potential in broader risk stratification and personalized treatment approaches.

## 17. Limitations

Several limitations in this review should be acknowledged. First, our evaluation was limited to studies published after 2019, which may have excluded valuable insights from earlier research on CAD biomarkers. Also, inclusion criteria nearly focused on chronic CAD patients, intentionally removing acute cardiac conditions. Though supporting a detailed analysis of chronic CAD biomarkers, this issue limits the broader applicability of our findings across the full CAD continuum. A quantitative assessment like meta-analysis would be helpful for assessing the efficacy of different biomarkers. Lastly, due to limitations in the available data and the scope of this review, we did not develop a predictive model for measuring CAD risk and its progression to adverse outcomes.

## 18. Conclusion

This review comprehensively examined a range of biomarkers that have been evaluated so far in recent years. Although certain biomarkers such as PTX3 and IL-37 have demonstrated high sensitivity and specificity, there is still a long way from research to the bedside. These biomarkers should be used cautiously through highly sensitive detection assays with confirmed reproducibility and reliability. Considering complexity of CAD pathology, these biomarkers serve various biological functions and belong to different categories, and hence, it is impractical to rely on a single molecule for early diagnosis. Moreover, although detection of such biomarkers is feasible through routine laboratory tests, it is beneficial to fine-tune such measurements by advancements in laboratory techniques, which improves sensitivity and specificity. Future prospective cohort and randomized controlled trials can be instrumental in identifying biomarkers that enable early and accurate CAD diagnosis.

## Author contributions

**Conceptualization:** Pouria Azami, Iman Razeghian-Jahromi.

**Data curation:** Pouria Azami, Soroush Seirafi.

**Investigation:** Soroush Seirafi.

**Methodology:** Soroush Seirafi.

**Project administration:** Iman Razeghian-Jahromi.

**Supervision:** Sahand Mohammadzadeh.

**Validation:** Sahand Mohammadzadeh.

**Visualization:** Pouria Azami, Sahand Mohammadzadeh, Iman Razeghian-Jahromi.

**Writing – original draft:** Pouria Azami.

**Writing – review & editing:** Pouria Azami, Soroush Seirafi, Iman Razeghian-Jahromi.
